# Brillouin-Raman mapping of natural fibers with spectral moment analysis

**DOI:** 10.1364/BOE.10.001469

**Published:** 2019-02-28

**Authors:** Daniele Fioretto, Silvia Caponi, Francesca Palombo

**Affiliations:** 1 Dipartimento di Fisica e Geologia, Università di Perugia, Via A. Pascoli, I-06100 Perugia, Italy; 2 Institute of Materials, National Research Council (IOM-CNR), Unit of Perugia, c/o Department of Physics and Geology, University of Perugia, Perugia, Italy; 3 School of Physics and Astronomy, University of Exeter, Stocker Road, EX4 4QL Exeter, United Kingdom

## Abstract

Brillouin spectroscopy has emerged as a novel analytical tool for biophotonic research and applications. It operates on a microscopic scale and in the GHz spectral range, providing a new spatial and frequency window for the analysis of the materials elasticity. Here we investigate spectral moments calculation as a means of analysing Brillouin and Raman spectra, providing rapid access to peak intensity and frequency shift, with robust application to fast scanning measurements. This work demonstrates the potential of the method, especially in the case of micro-structured samples, typical of bio-medical applications.

## 1. Introduction

In the last decades, optical methods gained a growing interest in the biomedical field: their label-free and contactless character allows the in situ analysis also offering a multi-modal approach. Recently, in addition to the chemical composition, also the physical characteristics have been recognized as particularly promising diagnostic markers for medical applications [1]. At macroscopic length scales, the biomechanical changes in tissues and organs are symptoms or effects of disease: probing the tissues biomechanics, important diagnostic results have been validated in ophthalmology and oncology [1–3]. Also at the microscopic level the mechanical characteristics play a key role: the elasticity of the microenvironment affects the behavior of cells and subcellular organelles [4,5], as well as cells change their elasticity depending on their healthy or pathogenic status [6–9]. Due to the large relevance of the mechanical properties at the cell and tissue level, Brillouin spectroscopy seems to be a strategic analytical tool with potential diagnostic capabilities. Its ability, widely exploited in material science [10–13], recently found new application areas in biology and biomedicine [3,14–20].

Strategies to optimize and standardize the Brillouin data analysis have to be refined in order to obtain, using the simplest approach, a robust and informative characterization of the elastic properties in structured and heterogeneous materials. The present study performed using a recently developed Brillouin-Raman system [19,21] presents the characterization of a composite sample made of natural fibers to validate a fast and reliable data analysis method based on calculation of zeroth and first order spectral moments.

## 2. Methods

### 2.1 Setup and sample

**Fig. 1:**
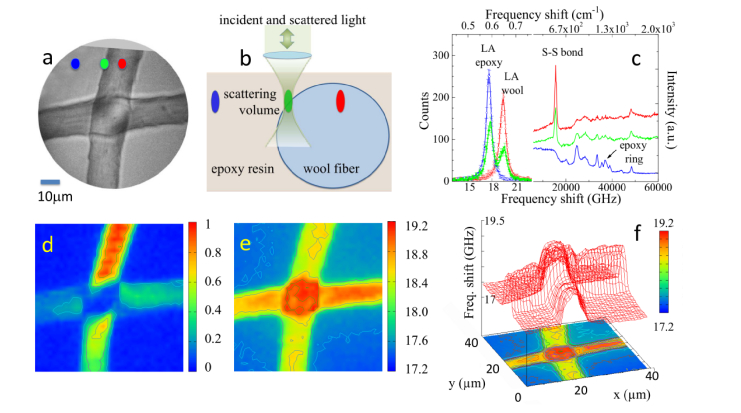
(a) Photomicrograph of wool fibers embedded into a 30 μm-thick epoxy film (DGEBA-DETA 5:2) mounted onto a reflecting silicon substrate. (b) Schematic diagram showing the focusing optics and scattering volumes for three points. (c) Brillouin spectra (left) and Raman spectra (right) extracted from the maps; they are color coded to the locations indicated in (a) and (b). (d) Raman map based on the normalized integrated intensity of the cysteine S–S band at 513 cm^–1^. (e) Brillouin map based on the average frequency shift (GHz) of the Brillouin peaks calculated from Eq. (1). (f) 3-D plot of $\bar{\nu }$mimicking the sample microstructure.

The experimental set-up enables to perform simultaneous micro-Brillouin-Raman mapping [19–21] using a tandem multipass Fabry-Pérot interferometer (HC-TFP -Table Stable) and a Raman spectrometer (Horiba iHR320 Triax). The sample here analyzed is a micro-structured composite system formed by wool fibers, of approximately 15 µm in diameter, embedded into a 30 µm-thick epoxy film mounted onto a reflective silicon substrate (Fig. 1(a)

). This model sample can be used: i) to test the capability of our technique to map differential elastic and chemical properties at microscales in composite materials, and ii) to prove the ability of the proposed data analysis in a simple micro-structured system.

### 2.2 Spectral analysis

The Brillouin spectrum of a homogeneous material, in backscattering experimental configuration, contains two distinct peaks associated with the Stokes and anti-Stokes scattering process by longitudinal acoustic modes. Anti-Stokes peaks obtained in three points of our sample are reported in Fig.1c. In viscoelastic media, to correctly take into account attenuation processes, Brillouin peaks can be fitted by a damped harmonic oscillator (DHO) function convoluted with the instrumental function [14]. The real (*M*’) and imaginary (*M*”) parts of the longitudinal elastic modulus can then be obtained from the frequency shift (ω*_b_*) and linewidth (Γ*_b_*) of the peaks through the relationships: $M^{'}=\rho \omega_{b}^{2}/q^{2}$ and $M"=\rho {\text{ω}}_{b}{\text{Γ}}_{b}/q^{2}$, where ρ is the mass density of the sample, *q* = 2*nk_i_* the exchanged momentum in the backscattering configuration, *n* the refractive index of the material and *k_i_* the wavevector of the incident light. In some cases, especially for low attenuation, a Lorentzian function is used to approximate the DHO line shape, where peak maximum ω*_0_* and linewidth Γ*_0_* have to be suitably corrected to obtain those of the DHO [22]. Fitting each spectrum using a single DHO or Lorentzian function becomes inappropriate analyzing heterogeneous samples, where multiple peaks occur and heterogeneous broadening becomes non-negligible. This effect is particularly critical when the scale of the material heterogeneities is intermediate between that of the scattering volume (some microns) and the phonons wavelength *λ_p_* ~300nm [16,19] or when crossing interfaces within a sample. The method proposed here represents a very fast and reliable way to extract the intensity *I* and the average frequency shift$\bar{\nu }$ of Brillouin peaks and of the Raman bands based on the calculation of spectral moments. This method could be particularly effective in rapid 2-D and 3-D mapping, where the fitting procedure may become a bottleneck for the scanning velocity.

In details, for spectra sampled at equally spaced frequency intervals *Δν*, the zeroth and first order spectral moments respectively are:

$$\begin{array}{l} I=\sum_{i}I_{i};\;\bar{\nu }=\frac{\sum_{i}I_{i}\nu_{i}}{I} \end{array}$$ (1)

where$\nu_{i}$ is the frequency shift and $I_{i}$ the corresponding intensity in the spectrum. The sum is performed after subtraction of the background,$I_{i}=I_{i}^{tot}-B_{i}$, where$I_{i}^{tot}$is the measured intensity and $B_{i}$ the subtracted background, and extended over a reasonably large frequency range around the peaks, with intensity tending to zero each side. The area *A* of the peak is obtained as *A* = *I* Δν. Being the intensities statistically independent, the variances are given by the relationships:

$$\begin{array}{c} \sigma^{2}(I)=\sum_{i}{\left(\frac{\partial I}{\partial I_{i}}\right)}^{2}\sigma^{2}(I_{i})=\sum_{i}\sigma^{2}(I_{i});\;\\ \sigma^{2}(\bar{\nu })={\sum_{i}\left(\frac{\partial \bar{\nu }}{\partial I_{i}}\right)}^{2}\sigma^{2}(I_{i})=\sum_{i}{\left(\frac{\nu_{i}-\bar{\nu }}{I}\right)}^{2}\sigma^{2}(I_{i}) \end{array}$$ (2)

where $\sigma^{2}(I_{i})=I_{i}^{tot}$. In the case of negligible background, *σ^2^*(*I_i_*) = *I_i_*, and Eq. (2) becomes:

$$\sigma^{2}(I)=I;\;\sigma^{2}(\bar{\nu })={\sum_{i}\left(\frac{\nu_{i}-\bar{\nu }}{I}\right)}^{2}I_{i}$$ (3)

**Fig. 2:**
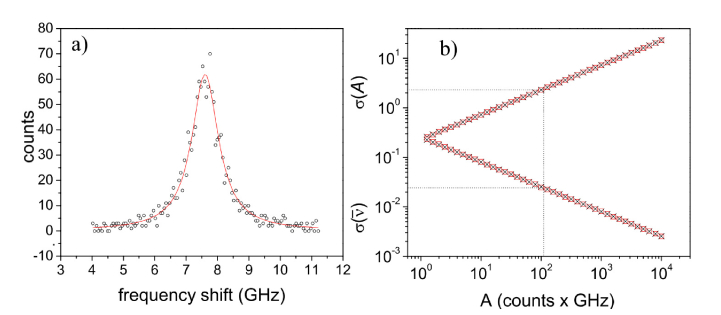
(a) Lorentzian curve with A = 102 counts·GHz (circles), *ν_0_* = 7.6 GHz and *Γ* = 1GHz, together with fit results obtained using a Lavembergue-Marquardt minimization routine (red line), giving *σ*(*ν_0_*) = 0.01 GHz and *σ*(*Α*) = 2 counts·GHz. (b) Calculated (black crosses, Eq. (3)) and simulated (red squares) variances associated with the intensity and average frequency.

Equations (2) and (3) are strictly correct in the case of very small variance of each *I_i_*. However, one can argue that the contribution of each *I_i_* is sufficiently small to make the application of Eqs. (2) and (3) plausible in a wide range of intensities. To check this hypothesis, we simulated Lorentzian peaks, centered at 7.6 GHz, with linewidth 1 GHz and amplitudes *A* ranging between 1 and 10^4^ counts·GHz. For each Lorentzian, we produced an ensemble of 10^4^ replicas with intensities *I_i_* modulated randomly by a Poissonian noise. As an example, Fig. 2(a)

shows a so simulated Lorentzian together with the fit results obtained using a Levemberg-Marquardt minimization routine [23]. Intensities and average frequencies were calculated for each simulated replica by Eq. (2) and the variances calculated for each ensemble are reported in Fig. 2(b), together with the values predicted through Eq. (3). The excellent agreement between calculated and simulated values corroborates the estimate of variances by means of Eqs. (2) and (3). Moreover, it is worth noting that the variance *σ*(*ν_0_*) obtained from fitting the sample Lorentzian in Fig. 2(a) is just about a factor 2 lower than that estimated by Eq. (3) (dotted lines in Fig. 2(b)). This is a quite reasonable result, since it is well known that the Levemberg-Marquardt method tends to underestimate variances.

These results confirm the ability of the proposed method, which can be used as regular basis for the analysis of symmetric (Lorentzian-like) Brillouin line-shapes, giving values for *I* and $\bar{\nu }$ with an accuracy similar to that obtained by least-square fitting procedures. Two additional arguments deserve to be mentioned emphasizing the convenience of this method and enlarging its field of applications. Firstly, it can be noticed that the values of relative intensity and average frequency shift of Lorentian-like Brillouin peaks are invariant after convolution with a symmetric instrumental response function. The effect of such convolution is just a symmetric broadening of the peak, so that the correct values for *I* and $\bar{\nu }$ can be obtained by applying Eq. (1) without the need for any deconvolution procedure.

Secondly, in the case of heterogeneous samples, when two or more sets of Brillouin peaks are simultaneously present in the spectrum, the simple property

$$\bar{\nu }=\frac{\sum_{i}(I_{1i}+I_{2i})\nu_{i}}{\sum_{i}I_{1i}+I_{2i}}=\frac{I_{1}{\bar{\nu }}_{1}+I_{2}{\bar{\nu }}_{2}}{I_{1}+I_{2}}$$ (4)

shows that the first spectral moment of the whole spectrum is the weighted average of the frequency position of the two peaks. In other words, even in the case of non-resolved (overlaid) peaks, calculation of the first spectral moment enables monitoring of the relative weight of the two contributions to be performed. This method, without resorting to sophisticated deconvolution procedures [19,24], was already used to estimate the relative modification of lipids vs. proteins content in Candida biofilms analyzing the high frequency Raman CH_2_, CH_3_ stretching band, which contains, superimposed, the contributions assigned to the two different chemical species [25]. More generally, in the heterogeneous multi-peak scenario, which frequently occurs in biomedical samples, Eq. (1) represents a suitable way to obtain the relevant information: the weighted-average of the different components. In the following, we show the ability of the method to estimate the relative volume fractions in a bi-component system.

## 3. Results

Figure 1(a) shows the photomicrograph of a sample made of two wool fibers embedded into an epoxy resin film (DGEBA-DETA 5:2). Each spectrum was collected in 10 seconds with an incident laser power of 5 mW. Longitudinal acoustic (LA) phonons propagating within the fibers give rise to Brillouin peaks at ~19 GHz, and those of the polymerized epoxy resin at ~17.5 GHz. A good chemical signature of wool fibers is given by the Raman peak near 513 cm^–1^, assigned to the disulfide bond that is abundant within the double amino acid cysteine [26], whilst the band near 1255 cm^–1^ is assigned to the epoxy ring stretching vibration [27].

The pseudo-color image in Fig. 1(d), obtained by raster-scanning the sample with 2μm step, 40 × 40 points, is a truly chemical map of the sample since each pixel corresponds to the intensity of the S–S stretching Raman peak, calculated by Eq. (1) after background subtraction. Here, it can be seen that the upper part of the vertical fiber, where the scattering volume better matches the surface of the fiber, is characterized by a very strong scattered intensity from disulfide bond, which is typical of cuticle cells. In fact, these epithelial cells, which form a protective layer of scales, are characterized by a higher content of sulfur with respect to the inner part of the fiber.

The mechanical map of the sample (Fig. 1(e)), i.e. the map of the average frequency$\bar{\nu }$ of the Brillouin peaks (calculated from Eq. (1)) was obtained simultaneously to the Raman map. The maximum in $\bar{\nu }$ of about 19 GHz is reached in the crossing area between the fibers, where the epoxy is largely excluded from the scattering volume. A small gradient in the frequency of LA modes of the epoxy resin is also visible away from the fibers, possibly due to a slightly heterogeneous mixing of DGEBA-DETA compounds. But the most interesting feature is the well-defined change in $\bar{\nu }$at the interface between resin and the wool fiber. In this region, the scattering volume is partially filled by the epoxy and partially by the wool, the classical origin of heterogeneities in Brillouin spectra. It is interesting to notice that, when the Brillouin scattering cross sections of the two components are comparable, the gradient in $\bar{\nu }$ reflects the gradient in the relative volume fractions. This effect is emphasized by the 3D representation in Fig. 1(f), where the map of Brillouin shift $\bar{\nu }$ mimics the volume fraction occupied by the wool fiber. Notice that this elaboration procedure is able to reproduce the morphology of the sample up to the detail relative to the relative positions of the two crossing fibers.

## 4. Conclusions

In conclusion, we have proposed a method based on the calculation of the zeroth and the first order spectral moments to obtain a fast and robust analysis of Brillouin and Raman spectral features also in case of heterogeneous samples. The variances in the values of intensity and average frequency obtained by this method are comparable with those obtained by standard non-linear least squares fitting routines. This method is thus particularly suitable for 2D and 3D mapping applications, where the rate of data analysis may be a limiting factor. Moreover, using spectral moments there is no need for selection of specific spectral functions that would not be normally applicable to heterogeneously broadened peaks. Intensity and frequency shift obtained by the zeroth and the first spectral moments simply give the average mechanical properties of matter within the analyzed scattering volume, truly a useful piece information in biomedical applications.
